# A bispecific enediyne-energized fusion protein targeting both epidermal growth factor receptor and insulin-like growth factor 1 receptor showing enhanced antitumor efficacy against non-small cell lung cancer

**DOI:** 10.18632/oncotarget.15933

**Published:** 2017-03-06

**Authors:** Xiao-Fang Guo, Xiao-Fei Zhu, Hai-Ying Cao, Gen-Shen Zhong, Liang Li, Bao-Guo Deng, Ping Chen, Pei-Zhen Wang, Qing-Fang Miao, Yong-Su Zhen

**Affiliations:** ^1^ Department of Microbiology, School of Basic Medical Sciences, Xinxiang Medical University, Xinxiang, China; ^2^ Department of Clinical Immunology, School of Laboratory Medicine, Xinxiang Medical University, Xinxiang, China; ^3^ Henan Collaborative Innovation Center of Molecular Diagnosis and Laboratory Medicine, Xinxiang, China; ^4^ Laboratory of Cancer Biotherapy, Institute of Neurology, The First Affiliated Hospital of Xinxiang Medical University, Weihui, China; ^5^ Department of Oncology, Institute of Medicinal Biotechnology, Chinese Academy of Medical Sciences and Perking Union Medical College, Beijing, China

**Keywords:** EGFR, IGF-1R, lidamycin, bispecific fusion protein, NSCLC

## Abstract

Epidermal growth factor receptor (EGFR) and insulin-like growth factor 1 receptor (IGF-1R) both overexpressed on non-small cell lung cancer (NSCLC) and are known cooperatively to promote tumor progression and drug resistance. This study was to construct a novel bispecific fusion protein EGF-IGF-LDP-AE consisting of EGFR and IGF-IR specific ligands (EGF and IGF-1) and lidamycin, an enediyne antibiotic with potent antitumor activity, and investigate its antitumor efficacy against NSCLC. Binding and internalization assays showed that EGF-IGF-LDP protein could bind to NSCLC cells with high affinity and then internalized into cells with higher efficiency than that of monospecific proteins. *In vitro*, the enediyne-energized analogue of bispecific fusion protein (EGF-IGF-LDP-AE) displayed extremely potent cytotoxicity to NSCLC cell lines with IC_50_<10^−11^ mol/L. Moreover, the bispecific protein EGF-IGF-LDP-AE was more cytotoxic than monospecific proteins (EGF-LDP-AE and LDP-IGF-AE) and lidamycin. *In vivo*, EGF-IGF-LDP-AE markedly inhibited the growth of A549 xenografts, and the efficacy was more potent than that of lidamycin and monospecific counterparts. EGF-IGF-LDP-AE caused significant cell cycle arrest and it also induced cell apoptosis in a dosage-dependent manner. Pretreatment with EGF-IGF-LDP-AE inhibited EGF-, IGF-stimulated EGFR and IGF-1R phosphorylation, and blocked two main downstream signaling molecules AKT and ERK activation. These data suggested that EGF-LDP-IGF-AE protein would be a promising targeted agent for NSCLC patients with EGFR and/or IGF-1R overexpression.

## INTRODUCTION

Lung cancer has been the leading cause of cancer death worldwide. Non-small cell lung cancer (NSCLC) accounts for approximately 85% of all lung cancers, with this encompassing the pathologically distinct adenocarcinoma, squamous cell carcinoma, and large cell carcinoma types. Despite recent advances in chemotherapy, the global mortality rate of unresectable or metastatic NSCLC remains high, and the 5-year overall survival rate is less than 15% [1–3].

Epidermal growth factor receptor (EGFR) and insulin-like growth factor 1 receptor (IGF-1R) have been identified as promising therapeutic targets in NSCLC. EGFR, a member of ErbB receptor tyrosine kinase family, is known to play important roles in promoting cell survival, proliferation, differentiation, migration and angiogenesis when activated by ligand (EGF, TGF-α, etc) binding [4]. Cetuximab, gefitinib, erlotinib and afatinib, these monoclonal antibodies (mAbs) or small tyrosine kinase inhibitors (TKIs) which targeting EGFR have shown improved survival for patients with NSCLC compared with standard chemotherapy [5–8]. But tumors generally develop resistance to them within a few months, thereby limits the clinical efficacy of monospecific targeted therapy [9–11]. Results from many studies indicated that cross-talk between EGFR and IGF-1R may be one of the reasons for acquired resistance against EGFR-targeted drugs [12–15]. Insulin-like growth factor (IGF) signaling system which consisted of ligands (IGF-1 and IGF-2), growth factor receptors (IGF-1R, IGF-2R) and IGF binding proteins (IGFBPs 1-6), also has been implicated in the development, maintenance, and progression of cancer [16]. IGF-1 and IGF-2 are structurally related to insulin and they play a role in regulating cell growth, proliferation, transformation, differentiation, migration, and apoptosis. The physiological activities of IGF-1 and IGF-2 are modulated by their association with IGFBPs [17]. IGF-1R is a glycoprotein composed of two extracellular α subunits that bind IGF-1 preferentially and with lesser affinity to IGF-2 and insulin. The two β subunits contain the tyrosine kinase domain responsible for activation the two main downstream signaling pathways (PI3K/AKT pathway and Ras/MAPK pathway) that promote cell growth, transformation, migration, and survival [12, 16, 17]. Therapeutic strategies targeting IGF-1R, including the use of mAbs, TKIs, and IGF ligand neutralizing antibodies have been explored in preclinical studies. They inhibited the growth of IGF-IR expressing tumor cells *in vitro* and *in vivo*, and enhance responses of cancer cells to treatments with cytotoxic drugs or radiotherapy [18–25].

Overexpression of EGFR has been observed in a number of solid tumors, including 40% to 80% of NSCLC [26, 27]. High IGF-1R expression was also occurred in NSCLC and was associated with poor survival, and elevated plasma levels of IGF-1 have been associated with an increased risk of the disease [28]. Furthermore, a study has revealed that overexpression of both EGFR and IGF-1R was observed in 24.8% of 125 surgical NSCLC patients, and high co-expression of EGFR and IGF-1R was a significant prognostic factor of worse disease-free survival (DFS) [29]. A number of bispecific antibodies targeting both EGFR and IGF-1R (EI-04, XGFR) have demonstrated superior antitumor activity to corresponding monospecific antibodies [30–32], but the researches on ligand-based EGFR/IGF-1R bispecific fusion protein have not been reported yet. Antibody-drug conjugates or ligand-toxin fusion proteins taking advantage of the specificity of antibodies or ligands and the potent cytotoxic activity of toxins are an emerging novel class of anticancer treatment agents. Trastuzumab emtansine (T-DM1) consisting of trastuzumab coupled to a cytotoxic agent, emtansine (DM1), and DAB(389)IL-2 (denileukin diftitox, ONTAK) made up of the full-length IL-2 molecule and the catalytic domain of diphtheria toxin have approved by FDA and shown significant efficacy and safety in treating patients with advanced breast cancer and cutaneous T-cell lymphoma, respectively [33, 34].

In this study, we firstly report the construction and functional characterization of a ligand-based EGFR/-IGF-1R bispecific fusion protein EGF-IGF-LDP-AE. As shown, EGF and IGF-1, the natural ligands with high affinity to EGFR and IGF-1R were used as targeting moiety, and an enediyne antibiotic, lidamycin, was used as cytotoxic payload. Lidamycin (C-1027) showed extremely potent cytotoxicity to various cancer cells and exhibited marked inhibitory effects on a panel of xenografts in athymic mice. Lidamycin consists of two moieties, an active enediyne chromophore (AE) responsible for the extremely potent cytotoxicity and a noncovalently bound apoprotein (LDP), which forms a hydrophobic pocket for protecting the chromophore. The apoprotein and chromophore can be dissociated and reconstituted *in vitro* [35]. Thus, EGF and IGF-1 were fused to the LDP to obtain the fusion protein EGF-IGF-LDP firstly, and then the AE of lidamycin was integrated into the EGF-IGF-LDP to prepare the enediyne-energized fusion protein EGF-IGF-LDP-AE. This research measured the antitumor activity of EGF-IGF-LDP-AE on NSCLC, and compared the potency of bispecific fusion protein with lidamycin and its monospecific counterparts, EGF-LDP-AE and LDP-IGF-AE. The aim of this study was to confirm whether a bispecific fusion protein targeting both EGFR and IGF-1R offers a superior antitumor efficacy against NSCLC.

## RESULTS

### Construction, expression and purification of fusion proteins and their enediyne-energized analogues

**Figure 1:**
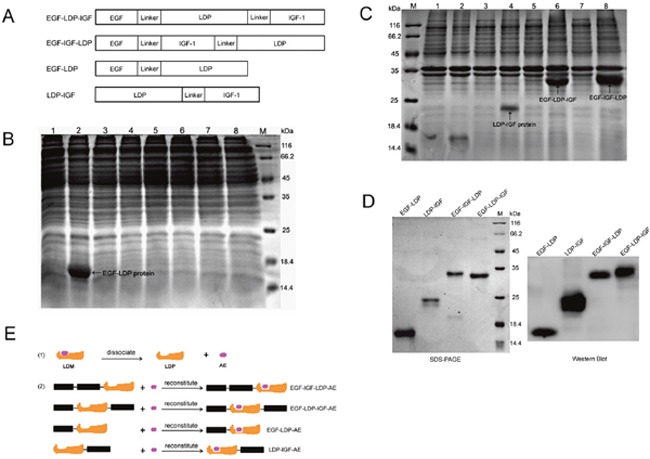
Construction, expression and purification of fusion proteins and their enediyne-energized analogues **(A)** diagram of genes encoding for fusion protein EGF-LDP-IGF, EGF-IGF-LDP, EGF-LDP and LDP-IGF. **(B, C)** SDS-PAGE analysis of soluble cytoplasmic proteins **(B)** or insoluble proteins **(C)** extracted from *E.Coli* carrying pET30-*egf-ldp*, pET30-*ldp-igf*, pET30-*egf-ldp-igf* and pET30-*egf-igf-ldp*, respectively. M, protein marker; Lane 1, 3, 5, 7 indicated proteins extracted from *E.Coli* carrying pET30-*egf-ldp*, pET30*-ldp-igf*, pET30*-egf-ldp-igf and* pET30*-egf-igf*-*ldp* before IPTG induction, respectively. Lane 2, 4, 6, 8 indicated proteins extracted from *E.Coli* carrying pET30-*egf-ldp*, pET30*-ldp-igf*, pET30*-egf-ldp-igf and* pET30*-egf-igf*-*ldp* after IPTG induction, respectively. **(D)** SDS-PAGE and Western blot analysis of the purified fusion proteins. **(E)** diagram of preparation of enediyne-energized fusion proteins EGF-IGF-LDP-AE, EGF-LDP-IGF-AE, EGF-LDP-AE and LDP-IGF-AE.

The DNA sequence coding for bispecific protein EGF-IGF-LDP (from 5’-end to 3’-end) consisted of human *egf* gene (159 bp), (GGGGS)_2_ linker (30 bp), human *igf-1* gene (210 bp), (GGGGS)_2_ linker (30 bp) and gene of apoprotein of lidamycin (*ldp*, 330 bp). Another bispecific fusion protein EGF-LDP-IGF that differed in the location of the *ldp* from EGF-IGF-LDP was constructed for comparison. Monospecific EGF-LDP protein which contains human *egf* gene, (GGGGS)_2_ linker and *ldp* gene, and LDP-IGF protein which contains *ldp* gene, (GGGGS)_2_ linker, and human *igf-1* gene were also synthesized (Figure 1A). The results from protein localization analysis revealed that EGF-LDP was soluble protein whereas EGF-IGF-LDP, EGF-LDP-IGF and LDP-IGF proteins were located in the insoluble fractions (Figure 1B, 1C). A (His)_6_-tag was introduced at the COOH terminal of all the fusion proteins, therefore, they were purified by Ni^2+^ affinity chromatography. The purity of fusion proteins were all over 90% when analyzed by SDS-PAGE (Figure 1D), and the production of EGF-LDP-IGF, EGF-IGF-LDP, EGF-LDP, and LDP-IGF was 36, 50, 53 and 12 mg/L fermentation broth, respectively. The enediyne-energized analogues of fusion proteins (EGF-IGF-LDP-AE, EGF-LDP-IGF-AE, EGF-LDP-AE and LDP-IGF-AE) were prepared by integrating AE molecule of lidamycin into the four fusion proteins (Figure 1E).

### Binding affinity and internalization efficiency of fusion proteins to NSCLC cells

**Figure 2:**
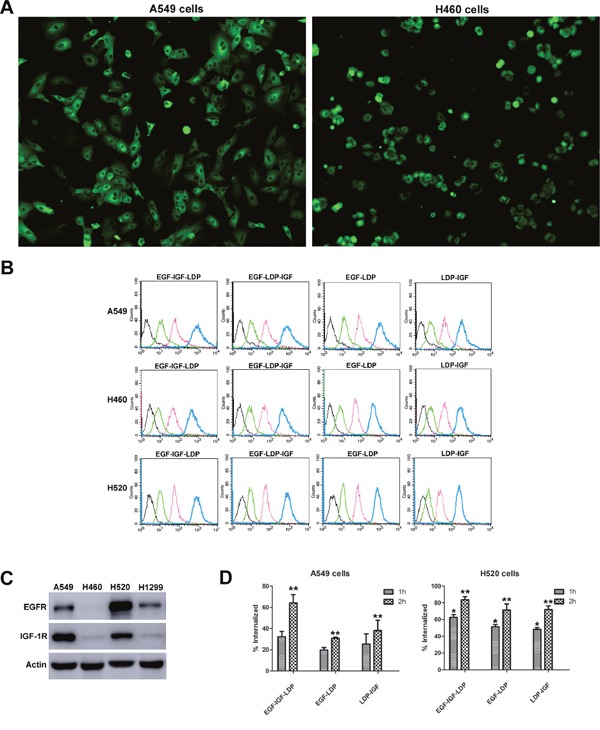
Binding and internalization of fusion proteins to NSCLC cells **(A)** EGF-IGF-LDP protein bound to the A549 and H460 cells detected by immunofluorescence assay. The images were observed under a fluorescence microscope at ×200. **(B)** FITC-labeled fusion proteins at different concentrations (black curve indicated the control groups, green, pink, and blue curves indicated the fusion proteins at 10 nmol/L, 100 nmol/L, and 1 μmol/L, respectively) were incubated with NSCLC cells and the mean fluorescence intensities were measured by a flow cytometer. **(C)** expression levels of EGFR and IGF-1R on different NSCLC cells analyzed by Western blot. **(D)** the internalization efficiencies of bispecific and monospecific fusion proteins when incubated with A549 and H520 cells at 37°C for 1 h or 2 h. *p < 0.05 (EGF-IGF-LDP vs EGF-LDP or LDP-IGF). **p < 0.01 (EGF-IGF-LDP vs EGF-LDP or LDP-IGF).

The results from immunofluorescence stain assay showed that EGF-IGF-LDP protein could bind to the NSCLC cells A549 and H460 (Figure 2A). A flow cytometry-based binding assay was also done to quantitatively compare the binding affinity of each fusion protein to NSCLC cells. A549, H460 and H520 cells were incubated with increasing concentrations of FITC-labeled fusion proteins, and the mean fluorescence intensities (MFIs) correspondingly increased (Figure 2B). Furthermore, the MFIs were linear with the concentration of FITC-labeled fusion proteins within the range of 0 nmol/L - 1 μmol/L (Supplementary Figure 1). Monospecific EGF-LDP protein and bispecific EGF-LDP-IGF, EGF-IGF-LDP protein showed similar high affinities to NSCLC cells, whereas LDP-IGF showed a significantly decreased affinity. For example, in H520 cells, the concentrations of EGF-LDP/FITC, EGF-LDP-IGF/FITC and EGF-IGF-LDP/FITC were 159.7 nmol/L, 165.5 nmol/L and 120 nmol/L, respectively, when the MFIs=100. However, the concentration of LDP-IGF/FITC was 449.8 nmol/L when the MFI=100, which is 3.7 times that of EGF-IGF-LDP. To determine whether the binding affinity of fusion proteins to NSCLC cells was correlated with the EGFR and IGF-1R expression levels, the expression of EGFR and IGF-1R on NSCLC cells was detected by Western blot analysis (Figure 2C), and two-way analysis of variance (ANOVA) and Bonferroni post-tests were carried out among the MFIs of A549, H460 and H520 cells (Prism 5 software). The results showed that the MFIs of EGF-IGF-LDP to A549 and H520 cells with high levels of EGFR and IGF-1R was significantly higher than that of H460 cells with low EGFR and IGF-1R expression (p < 0.05, Supplementary Figure 2). This proved that the binding affinity of the EGF-IGF-LDP protein to NSCLC cells was related to EGFR and IGF-1R expression levels.

From the results of flow cytometry-based internalization assay, we found that when incubated with A549 or H520 cells at 37°C for 1 h or 2 h, more EGF-IGF-LDP protein was internalized into cells than that of EGF-LDP and LDP-IGF proteins (Figure 2D). The increased internalization efficiency of bispecific EGF-IGF-LDP protein may contribute to its enhanced cytotoxicity.

### Cytotoxicity of fusion proteins and their enediyne-energized analogues to NSCLC cells

**Figure 3:**
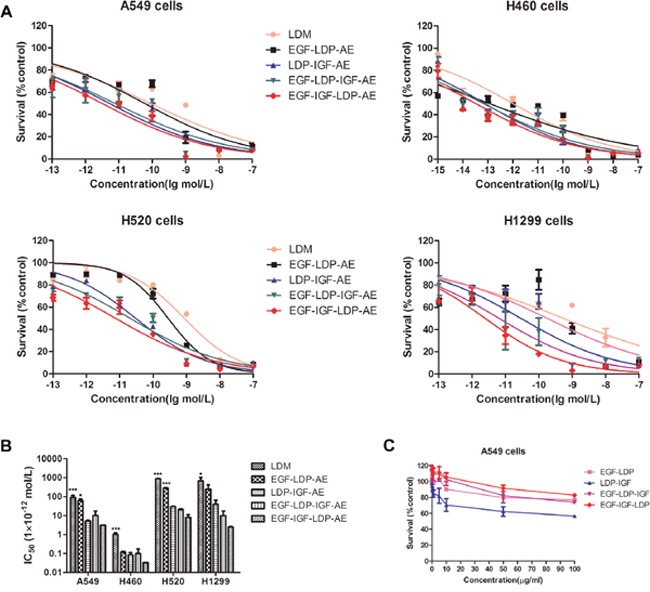
(A) cytotoxicity of lidamycin and enediyne-energized fusion proteins EGF-IGF-LDP-AE, EGF-LDP-IGF-AE, EGF-LDP-AE and LDP-IGF-AE to NSCLC cells was measured by MTT assays **(B)** the IC_50_ values of lidamycin, EGF-LDP-AE, LDP-IGF-AE, EGF-LDP-IGF-AE and EGF-IGF-LDP-AE against NSCLC cells. *p < 0.05 (EGF-IGF-LDP-AE vs lidamycin, EGF-LDP-AE or LDP-IGF-AE) and ***p < 0.001 (EGF-IGF-LDP-AE vs lidamycin, EGF-LDP-AE or LDP-IGF-AE) analyzed by one-way ANOVA and Dunnett's multiple comparison test. **(C)** viability of A549 cells after treatment with fusion proteins without active enediyne measured by MTT assay. Lines, mean of triplicate experiments, bars, SD.

As shown in Figure 3A and 3B, the enediyne-energized analogues of four fusion proteins EGF-LDP-AE, LDP-IGF-AE, EGF-LDP-IGF-AE and EGF-IGF-LDP-AE displayed extremely potent cytotoxicity to NSCLC cell lines A549, H460, H520 and H1299. Moreover, two-way ANOVA analysis revealed that, except for the cytotoxicity between EGF-IGF-LDP-AE and LDP-IGF-AE in A549 cells, the bispecific proteins EGF-IGF-LDP-AE was more cytotoxic than monospecific proteins (EGF-LDP-AE and LDP-IGF-AE) and lidamycin (p < 0.05). However, because of the interactions between the fusion proteins and their concentration, one-way ANOVA and Dunnett's multiple comparison tests of the IC_50_ values revealed that there were significant differences between EGF-IGF-LDP-AE and EGF-LDP-AE in A549 and H520 cells (not significant in H460 and H1299 cells). The differences between EGF-IGF-LDP-AE and another monospecific fusion protein LDP-IGF-AE were not statistically significant for all 4 ESCC cell lines (Figure 3B). EGF-IGF-LDP-AE also showed more potent cytotoxicity than another bispecific fusion protein EGF-LDP-IGF-AE except in H460 cells. As a result, EGF-IGF-LDP-AE was used in the following experiments.

The fusion proteins EGF-LDP, EGF-LDP-IGF and EGF-IGF-LDP without active enediyne chromophore did not exhibit significant cytotoxicity to A549 cells even at 100 μg/mL. However, the LDP-IGF protein showed an inhibition rate of 37.6% and 43.4% at 50 μg/mL and 100 μg/mL, respectively (Figure 3C). MTT assays were also done comparing A549 cells treated with bispecific EGF-IGF-LDP-AE with cells treated with an equimolar concentration of mixed EGF-LDP-AE and LDP-IGF-AE to determine if the increased cytotoxicity of bispecific proteins was due to the presence of the EGF and IGF-1 on the same single-chain molecule. The result showed that bispecific EGF-IGF-LDP-AE was more potent than that of the mixture of EGF-LDP-AE and LDP-IGF-AE, but the difference was not significant (p > 0.05) (Supplementary Figure 3).

### Effects of bispecific enediyne-energized fusion protein EGF-IGF-LDP-AE on the cell cycle distribution and cell apoptosis

**Figure 4:**
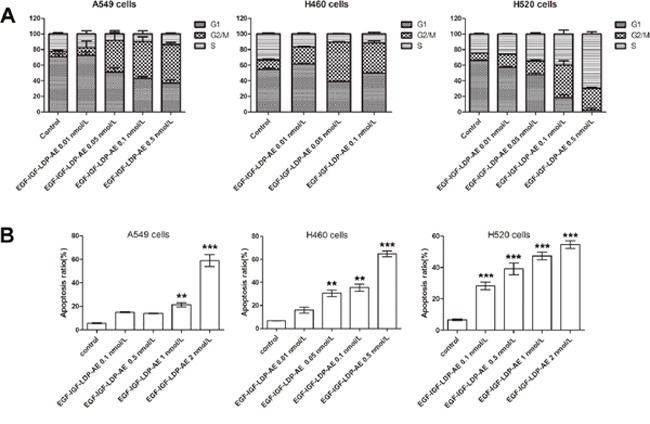
Effects of EGF-IGF-LDP-AE on cell cycle distribution and cell apoptosis **(A)** NSCLC cells were exposed to EGF-IGF-LDP-AE for 48 h at the indicated concentrations and cell cycle distribution was determined by flow cytometry after PI stain. **(B)** NSCLC cells were treated by EGF-IGF-LDP-AE for 48 h at the indicated concentrations and the apoptotic cells were stained by Annexin V-FITC and PI. Apoptosis ratios are the sum of early apoptotic cells and late apoptotic cells. **p < 0.01 (EGF-IGF-LDP-AE vs control). ***p < 0.001 (EGF-IGF-LDP-AE vs control).

After treatment with EGF-IGF-LDP-AE for 48 h, the cell cycle distribution of NSCLC cells altered significantly which the cells were arrested at G2/M phase. For example, control cells (A549) distributed in G2/M phase was 6.77% ± 1.8%, whereas cells treated with 0.01, 0.05, 0.1 and 0.5 nmol/L of EGF-IGF-LDP-AE distributed in G2/M phase were 9.73% ± 8.35%, 40.77% ± 9.99%, 47.09% ± 6.17% and 49.05% ± 2.98%, respectively. Similar results were also observed in H520 cells and H460 cells after exposure to EGF-IGF-LDP-AE (Figure 4A). But the G2/M phase cells reached the peak with 0.1 nmol/L of EGF-IGF-LDP-AE treatment for H520 cells, and with 0.05 nmol/L of EGF-IGF-LDP-AE treatment for H460 cells. The proportion of apoptotic and necrotic cells increased markedly after treatment with higher concentrations of EGF-IGF-LDP-AE, therefore the G2/M phase arrest was not significant as treatment with lower concentrations of EGF-IGF-LDP-AE.

Annexin V-FITC/PI staining assays revealed that EGF-IGF-LDP-AE treatment caused significant apoptosis of NSCLC cells in a dosage-dependent manner. As shown in Figure 4B, the ratios of apoptotic H520 cells after exposure to 0.1, 0.5, 1 and 2 nmol/L of EGF-IGF-LDP-AE were 28.27% ± 4.01%, 39.17% ± 6.45%, 47.38% ± 4.29%, and 54.6% ± 4.17%, respectively, which showed significant increases compared with control (6.6% ± 0.92%, p < 0.01). The apoptotic cells also increased a lot for the A549 cells and H460 cells after treatment with EGF-IGF-LDP-AE (Figure 4B).

### Effects of enediyne-energized fusion proteins on the EGFR/IGF-1R signaling pathways

**Figure 5:**
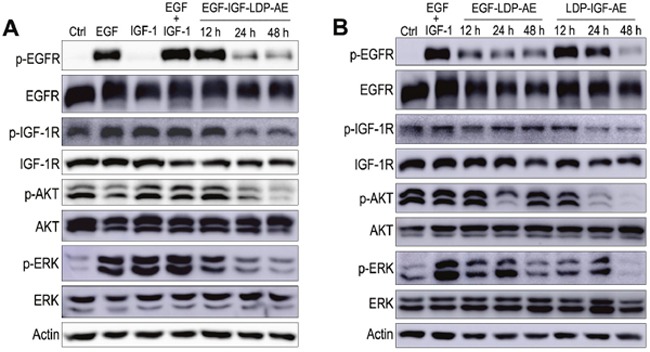
Effects of enediyne-energized fusion proteins on EGFR/IGF-1R signaling **(A)** A549 cells were treated with EGFIGF-LDP-AE (0.1 nmol/L) for 12 h, 24 h or 48 h, then the cells were stimulated with EGF or IGF-1 or IGF+IGF-1 for 30 min. And the phosphorylation of EGFR, IGF-1R, AKT, ERK and the total EGFR, IGF-1R, AKT, ERK were detected by Western blot analysis. Actin was used as a loading control. **(B)** phosphorylation and total expression levels of EGFR, IGF-1R, AKT and ERK were determined after exposure to monospecific fusion proteins (EGF-LDP-AE or LDP-IGF-AE) for 12 h, 24 h or 48 h by Western blot analysis.

EGFR and IGF-1R are two important receptor tyrosine kinases involved in cell growth and survival control by activating the two main signaling pathways: the PI3K-AKT pathway and Ras-MAPK pathway. Consequently, the effects of EGF-IGF-LDP-AE on the phosphorylation of EGFR and IGF-1R and the activation of two major downstream signaling molecules AKT and p44/p42 MAP kinases (ERK) were evaluated on A549 cells either stimulated or not with EGF and/or IGF-1. Incubation of A549 cells with EGF resulted in significant increased phosphorylation levels of EGFR, IGF-1R and ERK, while IGF-1 treatment could activate the IGF-1R, AKT and ERK. A significant increase in phospho-EGFR, phospho-IGF-1R, phospho-AKT and phospho-ERK was also observed in the presence of both EGF and IGF-1 (Figure 5A, lanes 2 - 4). Pretreatment with EGF-IGF-LDP-AE (0.1 nmol/L) for 12 h did not block EGF- and IGF-stimulated EGFR and IGF-1R phosphorylation, but their phosphorylation levels markedly reduced when exposure time extended to 24 h and 48 h. The activation of downstream signaling molecules AKT and ERK was also strongly inhibited by the treatment with EGF-IGF-LDP-AE for 24 h and 48 h. However, treatment with EGF-IGF-LDP-AE had no effect on the total expression levels of EGFR and IGF-1R as well as AKT and ERK (Figure 5A).

The effects of mono-specific fusion proteins EGF-LDP-AE and LDP-IGF-AE on the EGFR/IGF-1R signaling pathways were also evaluated. As shown in Figure 5B, A549 cells treated with EGF-LDP-AE for 12 h, 24 h and 48 h resulted in the inhibition of EGF- and IGF- stimulated phosphorylation of EGFR. LDP-IGF-AE pretreatment for 48 h could also inhibit the activation of EGFR. Phosphorylation of IGF-1R was not affected by the treatment of EGF-LDP-AE, but LDP-IGF-AE treatment for 24 h and 48 h caused the significant decrease of phospho-IGF-1R. Activation of AKT was suppressed when exposure to EGF-LDP-AE for 24 h and LDP-IGF-AE for 24 h, 48 h. Phospho-ERK was also reduced after exposure to EGF-LDP-AE for 48 h and LDP-IGF-AE for 12 h, 24 h, 48 h (Figure 5B). Similar with the bispecific EGF-IGF-LDP-AE protein, two mono-specific fusion proteins did not show any effects on the total levels of EGFR, IGF-1R, AKT and ERK.

### *In vivo* efficacy of enediyne-energized fusion proteins

**Figure 6:**
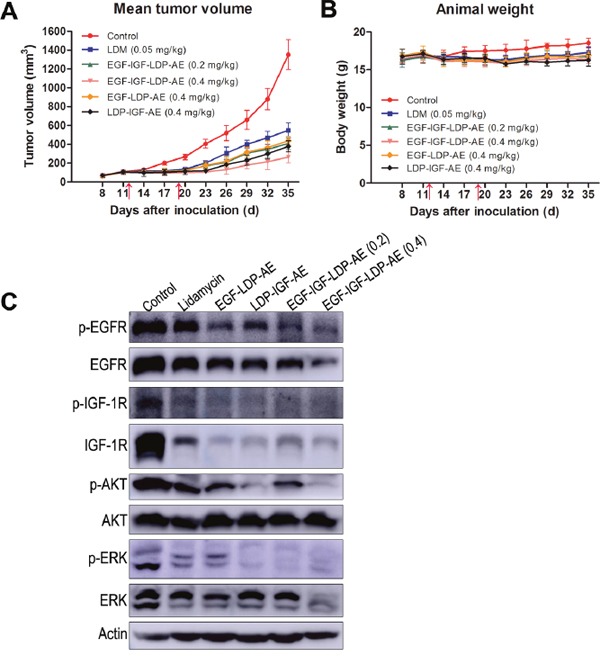
*In vivo* efficacy of lidamycin and enediyne-energized fusion proteins Nude mice (n = 6) bearing human NSCLC A549 xenografts were treated with lidamycin, EGF-IGF-LDP-AE, EGF-LDP-AE and LDP-IGF-AE through the tail vein injection, and the mean tumor volume **(A)** and animal weight **(B)** in each group were shown. The arrows indicated the time of injection (day 12 and day 19 after tumor inoculation). **(C)** the inhibitory effects on EGFR/IGF-1R signaling of lidamycin, EGF-LDP-AE, LDP-IGF-AE and EGFIGF-LDP-AE in xenograft tumors. Mice bearing A549 xenografts were sacrificed on day 35. The tumors were taken out from the mice and immediately frozen in liquid nitrogen. Total proteins were extracted from tumor tissues and the phosphorylation and total expression levels of EGFR, IGF-1R, AKT and ERK were evaluated by Western blot analysis.

The *in vivo* efficacy of bispecific EGF-IGF-LDP-AE and monospecific EGF-LDP-AE, LDP-IGF-AE were tested in an A549 xenografts nude mouse model. Mice bearing established xenografts of ∼100 mm^3^ were treated with lidamycin, EGF-LDP-IGF-AE, EGF-LDP-AE and LDP-IGF-AE twice by intravenous injections. EGF-IGF-LDP-AE at 0.2 mg/kg and 0.4 mg/kg both yielded significant tumor growth inhibition (69.7% and 80.5%, respectively; p < 0.01 compared with the PBS-treated group, p < 0.05 between two different dosages). Furthermore, the EGF-IGF-LDP-AE-treated group at 0.2 mg/kg and 0.4 mg/kg showed more potent growth inhibition when compared with lidamycin-treated group at maximum tolerated dosage (0.05 mg/kg, inhibition rate, 59.4%, p < 0.01). The monospecific enediyne-energized fusion proteins EGF-LDP-AE and LDP-IGF-AE (at 0.4 mg/kg) demonstrated similar antitumor activity (67.6% and 72% tumor growth inhibition, respectively, p > 0.05) to the bispecific fusion protein EGF-IGF-LDP-AE when given at 0.2 mg/kg. However, when given at same dosages (0.4 mg/kg), EGF-IGF-LDP-AE treatment resulted in the best antitumor activity among all the groups (Figure 6A; p < 0.01 compared with the EGF-LDP-AE-treated group and LDP-IGF-AE-treated group). During the experiment, no animal deaths were found, and Figure 6B showed that body weight loss resulting from the lidamycin, EGF-IGF-LDP-AE, EGF-LDP-AE and LDP-IGF-AE treatment at the termination of the experiment did not exceed 10% of the pretreatment weight. Thus, the animals tolerated well to the administered dosage of fusion proteins.

To further investigate the mechanisms of antitumor efficacy of fusion proteins *in vivo*, we analyzed the phosphorylation and total expression levels of EGFR, IGF-1R, AKT and ERK in xenograft tumor tissues by Western blot. The results demonstrated that phospho-EGFR reduced significantly in the EGF-LDP-AE, LDP-IGF-AE and EGF-IGF-LDP-AE (both 0.2 mg/kg and 0.4 mg/kg) treated tumors when compared with control group. Activation of IGF-1R, AKT and ERK was inhibited in lidamycin, EGF-LDP-AE, LDP-IGF-AE and EGF-IGF-LDP-AE (both 0.2 mg/kg and 0.4 mg/kg) treatment tumors. Besides, the total expression levels of EGFR, IGF-1R and ERK were also influenced by the treatment of fusion proteins. EGFR and ERK markedly reduced only in EGF-IGF-LDP-AE (0.4 mg/kg)-treated group and IGF-1R reduced significantly in all treated groups. But the total AKT remained unchanged in all groups (Figure 6C).

## DISCUSSION

Due to the primary and acquired resistance to the existing targeted drugs, such as monoclonal antibodies and tyrosine kinase inhibitors, there is an urgent need for developing new strategies against metastatic tumors. Antibody-drug conjugates (ADCs) and chimeric fusion proteins that combining the selectivity of targeted treatment with the cytotoxic potency of toxins, were highly effective in treating some of tumors. For example, trastuzumab emtansine (T-DM1), denileukin diftitox (DAB_389_IL2, Ontak) and RFB4 (dsFv)-PE38 (BL22) all showed improved antitumor efficacy in clinical trials [33, 34, 36, 37]. Despite these successful examples, immunotoxin or fusion protein has been associated with significant toxicities to normal tissues and limited anti-tumor efficacy for solid tumors in many cases [38–40]. A number of studies have revealed that targeting two tumor associated receptors would improve the selectivity over normal tissue that expresses only one (or low levels of both) target receptor [41–43]. The fusion protein EGF-IGF-LDP-AE we constructed in present study, is a bispecific molecule that targeting EGFR and IGF-1R. Both the EGFR and IGF-1R contributed to tumor development and progression through their effects on cell proliferation, apoptosis and angiogenesis, and both were overexpressed in NSCLC cells. As a result, bispecific enediyne fusion protein EGF-IGF-LDP-AE showed more potent cytotoxicity to NSCLC cells than that of monospecific counterparts (EGF-LDP-AE and LDP-IGF-AE) and naked lidamycin *in vitro*. EGF-IGF-LDP-AE was also able to more effectively inhibit the growth of A549 xenografts *in vivo*. Bispecific EGF-IGF-LDP-AE at 0.2 mg/kg and 0.4 mg/kg yielded tumor growth inhibition of 69.7% and 80.5%, respectively, whereas the lidamycin-treated group at maximum tolerated dosage (0.05 mg/kg) only showed inhibition rate of 59.4% (p < 0.01). Furthermore, EGF-IGF-LDP-AE showed more significant growth-inhibitory effects than that of the monospecific enediyne-energized fusion proteins EGF-LDP-AE and LDP-IGF-AE when given at same dosages (0.4 mg/kg, p < 0.01).

Different from the fully antibodies or the fragments of antibody (such as Fab, ScFv, etc) that were used as targeting molecules, the targeting moiety of EGF-IGF-LDP-AE protein is the two natural ligands: EGF and IGF-1. Firstly, EGF and IGF-1 has high affinity to their receptors with the Kd values of 1.77×10^−7^ mol/L and 4.45×10^−9^ mol/L, respectively [44, 45]. Secondly, the EGF and IGF-1 are both small proteins with molecular weights of 6.2 kDa and 7.6 kDa, respectively. And the cytotoxic moiety lidamycin (15 kDa) was also much smaller than the conventional toxins been used, such as truncated *Pseudomonas* exotoxin (PE40, 40 kDa), ricin A (32 kDa), and diphtheria toxin (DT389, 43 kDa) [46–48]. The resultant EGF-IGF-LDP protein had a molecular weight of 27.1 kDa, which would offer EGF-IGF-LDP protein improved solid tumor penetration and decreased immunogenicity.

Bound and internalization of EGF-IGF-LDP-AE to NSCLC cells was the prerequisite to exert its tumor cell killing effect. The immunofluorescence assay showed that EGF-IGF-LDP could bind to NSCLC cells with high affinity and then it internalize into the cells by receptor-mediated endocytosis. The internalization efficiency of EGF-IGF-LDP protein was significantly higher than that of monospecific proteins (EGF-LDP and LDP-IGF), and this may be one of the reasons for its enhanced cytotoxicity.

Chemotherapy drugs (such as doxorubicin and methotrexate) have been firstly used as cytotoxic payload to create ADCs. However, studies have shown that the actual concentration of these drugs in tumor cells is minimal with only 1% - 2% of the administered dose reaching the tumor [38, 49]. As a result, it is imperative to have a potent cytotoxic payload, being effective at picomolar or nanomolar concentrations. Lidamycin, used in this study as the cytotoxic moiety, was extremely cytotoxic to multiple cancer cells with IC_50_ values 1000-fold lower than that of mitomycin C and adriamycin [35]. In addition to the potent cytotoxicity, another attractive property of lidamycin being a “warhead” is that the apoprotein (LDP) and AE can be dissociated and reconstituted *in vitro*, which makes the construction of fusion proteins very convenient. Targeting molecules (such as ligands or scFvs) fused to the LDP firstly, then integrating with the AE to prepare the intact fusion proteins with high potency. Due to the above-mentioned two advantages, lidamycin has been widely used as an effector molecule (“warhead”) for the construction of fusion proteins. In previous studies, five bispecific and enediyne-energized fusion proteins with enhanced antitumor efficacy have been prepared and evaluated by our lab, such as EGFR/HER2-specific fusion protein Ec-LDP-Hr-AE, anti-gelatinases tandem scFv format dFv-LDP-AE, EGFR/CD13-targeting fusion protein ER(Fv)-LDP-NGR-AE, Ec-LDP-TRAIL which consisted of an oligopeptide against EGFR and tumor necrosis factor-related apoptosis-inducing ligand (TRAIL), and the tuftsin-based, EGFR-targeting fusion protein Ec-LDM-TF [50–54]. These bispecific fusion proteins demonstrated many advantages over monospecific counterparts, such as enhanced antitumor efficacy, prolonged tumor localization *in vivo*, elevated apoptosis-inducing effect and the immunostimulating effect.

Studies have reported that lidamycin inhibits DNA synthesis and causes cellular DNA breakage in cancer cells [35], EGF-IGF-LDP-AE also mainly exerted its cell-killing effect by damaging the DNA as single cell gel electrophoresis assay showed that a significant “comet tail” was observed after treatment with EGF-IGF-LDP-AE at 0.3 pmol/L and 3 pmol/L for 48 h (Supplementary Figure 4). Significant cell cycle arrest and apoptosis was observed after NSCLC cells exposure to EGF-IGF-LDP-AE. It also inhibited the activation of EGFR and IGF-1R, and the two major downstream signaling pathways, Ras/MAPK and PI3K/AKT. These mechanisms all contributed to the antitumor activity of EGF-IGF-LDP-AE.

In summary, this study firstly showed that the novel ligand-based EGFR/IGF-1R bispecific fusion protein EGF-IGF-LDP-AE not only demonstrated potent cytotoxicity to NSCLC cells *in vitro*, but also was highly effective in inhibiting the growth of A549 xenografts *in vivo*. The two receptor targeting property, potent antitumor efficacy and its much smaller molecular size, suggested that EGF-IGF-LDP-AE would be a promising candidate for NSCLC targeted therapy.

## MATERIALS AND METHODS

### Ethics statement

Animal experiment has been conducted in accordance with the ethical standards and according to the Declaration of Helsinki and according to national and international guidelines and has been approved by the Ethics Committee of Xinxiang Medical University.

### Cell lines and culture

Human non-small cell lung cancer cell line A549, NCI-H460, NCI-H520 and NCI-H1299 were obtained from Cell Bank of the Chinese Academy of Sciences (Shanghai, China) within 6 months of experiment. All cell lines were tested by the cell bank for viability (trypan blue staining), mycoplasma contamination, variation (DNA-Fingerprinting), interspecies contamination (iso-enzyme analysis), and endotoxin. All cells were cultured under a humidified atmosphere of 5% CO_2_ at 37°C in F12K (A549 cells) or RPMI1640 supplemented with 10% fetal bovine serum (Life Technologies), 100 U/mL penicillin and 100 μg/mL streptomycin.

### Construction, expression and purification of fusion proteins

DNA sequences coding for EGF-LDP-IGF and EGF-IGF-LDP were synthesized by Beijing Sunbiotech Co. Ltd. (Beijing, China), and then they were digested by *Nde*I/*Xho*I and were inserted into pET30a expression vector to generate plasmid pET30-*egf-ldp-igf* and pET30-*egf-igf-ldp*. Coding sequences for monospecific fusion protein EGF-LDP and LDP-IGF were created by the same way. DNA sequencing analysis (Invitrogen Corp.) was used to verify that the gene was correct in sequence and had been cloned in the frame.

Expression plasmids pET30-*egf-ldp-igf*, pET30-*egf-igf-ldp*, pET30-*egf-ldp* and pET30-*ldp-igf* were all transformed into *Escherichia coli* strain BL21(DE3) competent cells. For protein solubility analysis, single colony of pET30-*egf-ldp-igf*, pET30-*egf-igf-ldp*, pET30-*egf-ldp* and pET30-*ldp-igf* were inoculated into 100 mL LB medium containing 50 μg/mL kanamycin and grown at 37°C until the OD600 reaches 0.8. Just prior to induction, split the 100 mL culture into 2×50 mL cultures, add isopropyl-β-D-thiogalactopyranoside (IPTG) to one of the 50 mL cultures at 1 mmol/L and use the other culture as an uninduced control. Eight hours after induction, bacteria were harvested by centrifugation for 10 min at 10,000 g. The pellet was resuspended in 5 mL cold lysis buffer (20 mmol/L Tris-Hcl pH7.5, 0.5 mol/L NaCl, 5% glycerol) and sonicated on ice using a microtip at 40% duty for 30 min. Centrifuge the entire lysate at 14,000 g for 10 min to separate the soluble and insoluble fractions. The culture conditions that affect the expression level and solubility of recombinant proteins, such as growth temperature, cell density, concentration of IPTG and induction time were optimized to improve the yield.

The fusion proteins were purified by Ni^2+^ affinity chromatography (HisTrap HP, GE Healthcare) according to the manufacture's protocol. The insoluble proteins (EGF-LDP-IGF, EGF-IGF-LDP and LDP-IGF) were dissolved in the binding buffer including 8 mol/L urea and the purified denatured proteins were refolded in a way of stepwise dialysis as reported by Tsumoto *et al* [55].

### Binding affinity and internalization assays

Immunofluorescence stain assay and flow cytometry (FCM)-based binding assay were used to analyze the binding of fusion proteins to NSCLC cells. For immunofluorescence stain assay, A549 or H460 cells were grown on coverslides and cultured at 37°C for 24 h. Then cells were fixed with paraformaldehyde, blocked with normal goat serum, and incubated with EGF-IGF-LDP protein (100 μg/mL) for 2 h at room temperature. After three washes with PBS, cells were incubated with mouse anti-His-tag monoclonal antibody (TIANGEN Biotech, Beijing, China; diluted 1:100), followed with Alexa Fluor 488-conjugated goat anti-mouse antibody (Beyotime Biotechnology, Jiangsu, China; diluted 1:50). The images were observed under a fluorescence microscope (Nikon TE 2000u).

For FCM-based binding assay, all fusion proteins were FITC labeled as reported previously [50]. Each FITC-labeled fusion protein, EGF-LDP-IGF, EGF-IGF-LDP, EGF-LDP or LDP-IGF was incubated with 10^5^ A549, H520 or H460 cells in a 500 μL volume of PBS for 2 h at room temperature. Following three washes with 500 μL of PBS, cells were analyzed with flow cytometer (BD Company). FCM-based internalization assays were also done to compare the internalization efficiency of bispecific EGF-IGF-LDP and monospecific EGF-LDP and LDP-IGF as described by Stish *et al*. [56].

### Preparation of the enediyne-energized analogue of fusion proteins

We have previously found that the active enediyne chromophore (AE) of lidamycin could release from its hydrophobic pocket of LDP in organic solvent and, the detached LDP and AE can be reconstitute to form intact lidamycin in PBS buffer [35]. Therefore, the AE of lidamycin was separated by using C4 column (GE Healthcare) with a 22% acetonitrile in 0.05% trifluoroactic acid mobile phase, and then the AE-containing solution was added to EGF-LDP-IGF/PBS (10 mmol/L; pH7.4), EGF-IGF-LDP/PBS, EGF-LDP/PBS, and LDP-IGF/PBS, respectively, with the molecular ratio of 3:1 to prepare their enediyne-energized analogues EGF-LDP-IGF-AE, EGF-IGF-LDP-AE, EGF-LDP-AE and LDP-IGF-AE. Free AE was removed by using a Sephadex G-75 column (GE Healthcare).

### Cell viability assay

MTT assay was used to measure the effects of fusion proteins and its enediyne-energized analogues on the cell viability as described previously [50]. Briefly, NSCLC cell lines A549, H460, H520 and H1299 were seeded in 96-well plate and incubated at 37°C for 24 h. Then different concentrations of lidamycin or fusion proteins were added and incubated for 48 h. MTT solution (Sigma, 5 mg/mL, 20 μL) was added to each well and incubated for another 4 h at 37°C. The supernatant was removed and 150 μL Dimethyl Sulphoxide (DMSO) were added to each well. The absorbance at 570 nm was measured using an ELISA reader (Thermo Fisher). Growth inhibition was calculated as a percentage of the untreated controls.

### Cell cycle distribution analysis

A549, H520 and H460 cells were treated with 0.01 nmol/L, 0.05 nmol/L, 0.1 nmol/L, 0.5 nmol/L and 1 nmol/L EGF-IGF-LDP-AE for 48 h. Then the cells were harvested by trypsinization, washed with PBS, and fixed with cold 70% ethanol at -20°C for 24 h. Cells was centrifuged at 1,000 rpm for 5 min and the cell pellet was washed three times with PBS and resuspended in 0.5 mL PBS with 50 μg/mL propidium iodide (PI) and 100 μg/mL RNase A. After incubation at 37°C for 30 min, cells were analyzed for fluorescence with a flow cytometer (BD Company) and determined the percentage of cells in specific cell cycle phases (G1, S, and G2/M).

### Cell apoptosis assay

A549, H520 and H460 cells were seeded in the 6-well plate at the density of 10^5^ and incubated for 24 h. After treatment with 0.1 nmol/L, 0.5 nmol/L, 1 nmol/L and 2 nmol/L EGF-IGF-LDP-AE for 48 h, cells were harvested (including the non-adherent cells), washed twice with PBS, and stained with Annexin V-FITC and PI according to the manufacture's protocol (Beyotime Biotechnology, Jiangsu, China). The fluorescence intensity was measured with a flow cytometer (BD Company).

### Western blot analysis

A549 cells were plated into 100-mm dishes at the density of 5×10^5^ and grown to 70% - 80% confluence, after which the cells were washed three times in PBS and cultured for 12 h in serum free medium. Cells were incubated with EGF-IGF-LDP-AE (0.1 nmol/L), EGF-LDP-AE (0.1 nmol/L) or LDP-IGF-AE (0.1 nmol/L) at 37°C for 12 h 24 h or 48 h, followed by stimulation with EGF (Abcam, 50 ng/mL), IGF-1 (Abcam, 50 ng/mL), or both at 37°C for 30 min. The cells were lysed for 30 min in lysis buffer for Western blot and immunoprecipitation (Beyotime Biotechnology, Jiangsu, China) supplemented with 1 mmol/L phenylmethylsulfonyl fluoride (PMSF). For proteins extracted from tumor tissues, 100 mg tumor tissues were put in 1 mL RIPA lysis buffer (Beyotime Biotechnology, Jiangsu, China) supplemented with 1 mmol/L PMSF, homogenized on ice, and lysed on ice for 30 min. Extracts were clarified by centrifugation at 10,000 g for 15 min at 4°C, and total protein was quantitated using bicinchoninic acid kit (Pierce Biochemicals). 30 μg of each total protein were applied on 10% SDS-PAGE and then electroblotted onto polyvinylidene difluoride (PVDF) membranes (Millipore). The membranes were incubated with 1% BSA for 2 h at room temperature before incubation overnight at 4°C with primary antibodies (diluted 1:1000, Cell Signaling Technology). Then the membranes were incubated with secondary HRP-conjugated antibodies (1:3000 dilution; Cell Signaling Technology) for 1 h after washing three times with TBST buffer. The specific bands were visualized with the Immobilon Western Chemiluminescent HRP Substrate kit (Millipore) and captured by AI600 imaging system (GE Corp.).

### *In vivo* efficacy experiment

Female BALB/c nude mice (18 - 22 g) were purchased from VitalRiver Laboratory Animal Technology Co. Ltd., and hosted under specific pathogen-free conditions. 5×10^6^ A549 cells suspended in 200 μL PBS were inoculated s.c. in right armpit of nude mice. Tumor-bearing mice were randomly divided into groups (n = 6) when the tumor size was over 100 mm^3^, and treated with lidamycin, EGF-IGF-LDP-AE, EGF-LDP-AE, and LDP-IGF-AE, respectively, at different dosages. All fusion proteins were injected i.v. in the tail vein and given in a 200 μL volume of PBS. One week after the first treatment, tumor-bearing mice were injected with the fusion proteins again at same dosages. Tumor size was measured every 3 day and tumor volume was determined by a × b^2^/2, where a, and b indicated the long and the perpendicular short diameters of the tumor, respectively. The inhibition rates were calculated by 1 - tumor volume (treated)/tumor volume (control) ×100%.

## SUPPLEMENTARY MATERIALS FIGURES AND TABLES


