# Supplementary material for: A bispecific enediyne-energized fusion protein targeting both epidermal growth factor receptor and insulin-like growth factor 1 receptor showing enhanced antitumor efficacy against non-small cell lung cancer
Source: Oncotarget. 2017 Mar 6;8(16):27286–99. doi: 10.18632/oncotarget.15933 (PMC5432335; doi:10.18632/oncotarget.15933)
Supplement: Supplementary file 1 [file oncotarget-08-27286-s001.pdf]

A bispecific enediyne-energized fusion protein targeting both epidermal growth factor receptor and insulin-like growth factor 1 receptor showing enhanced antitumor efficacy against non-small cell lung cancer

SUPPLEMENTARY MATERIALS

SUPPLEMENTARY FIGURES

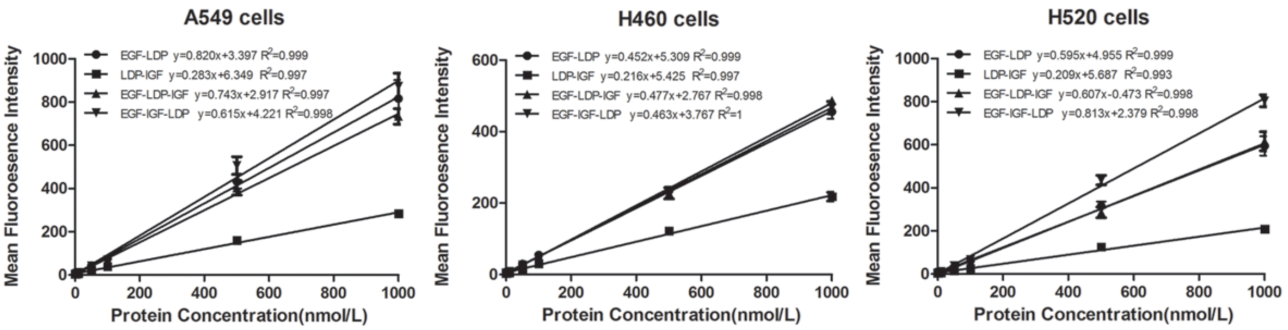

Supplementary Figure 1: The linear relationship between the mean fluorescence intensities (MFIs) and the concentration of FITC-labeled fusion proteins within the range 0 nmol/L to 1 µmol/L.

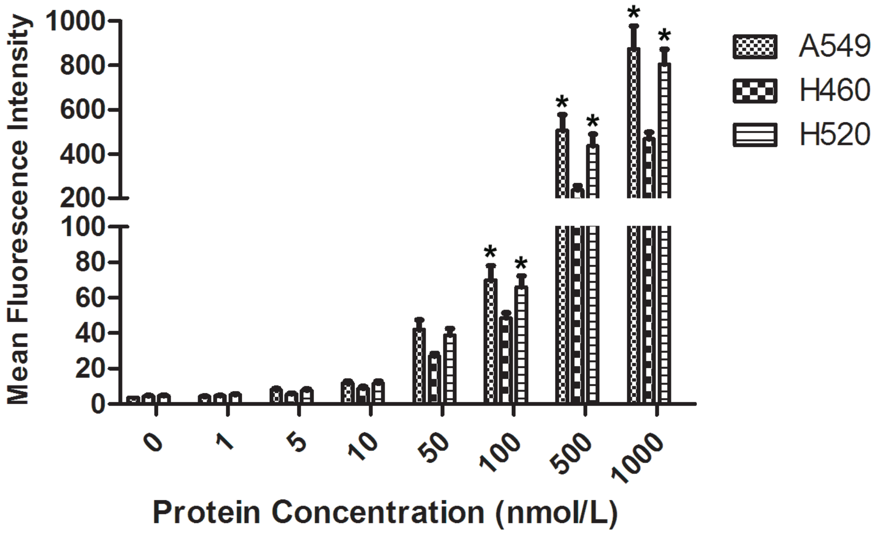

Supplementary Figure 2: The comparison of mean fluorescence intensities (MFIs) among A549, H460 and H520 cells. A549, H460 or H520 cells were incubated with increasing concentrations of FITC-labeled fusion proteins, and the MFIs of cells were measured by a flow cytometer after three washes with PBS. *$p < 0.05$, H460 vs A549 or H520.

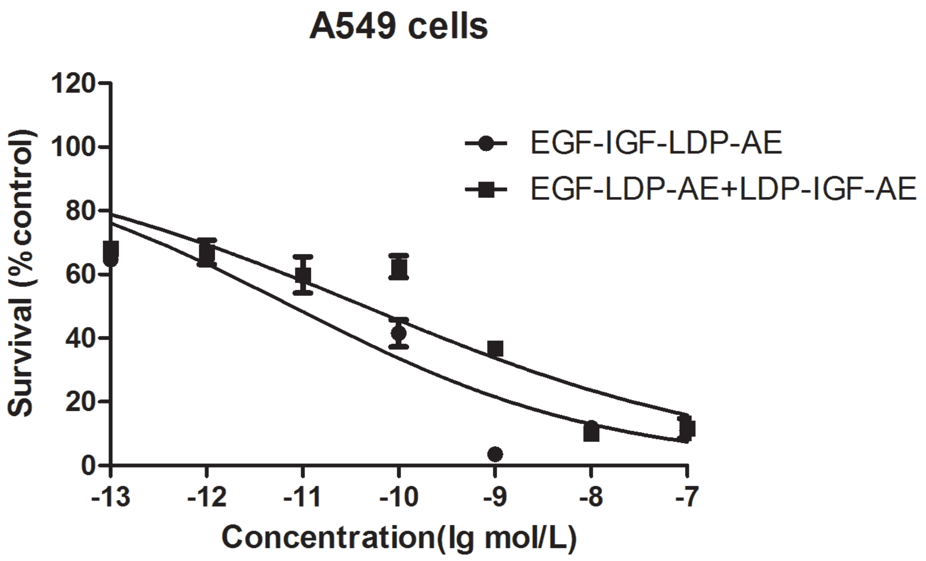

Supplementary Figure 3: MTT assay were done comparing A549 cells treated with EGF-IGF-LDP-AE to cells treated with a mixture of equimolar concentration of monospecific EGF-LDP-AE and LDP-IGF-AE. Lines, mean of triplicate experiments, bars, SD.

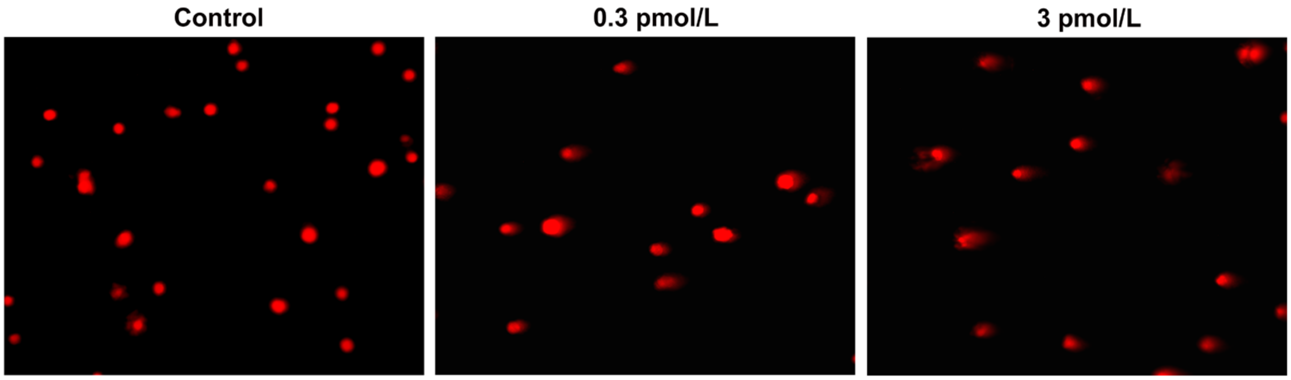

Supplementary Figure 4: The effect of EGF-IGF-LDP-AE on cell DNA damage measured by single cell gel electrophoresis assay. A549 cells were exposure to EGF-IGF-LDP-AE at 0.3 pmol/L and 3 pmol/L for 48 h, then single cells were embedded in a agarose gel on a microscope slide and lysed. The DNA was unwound in alkaline buffer, electrophoresed, and stained with propidium iodide, and the images were observed and captured by fluorescence microscope at ×400.
